# Comparative investigation of reusable and single–use flexible endoscopes for urological interventions

**DOI:** 10.1038/s41598-020-62657-w

**Published:** 2020-03-30

**Authors:** Maximilian Eisel, Frank Strittmatter, Stephan Ströbl, Christian Freymüller, Thomas Pongratz, Ronald Sroka

**Affiliations:** 1 Laser-Forschungslabor, LIFE-Zentrum, University Hospital of Munich, Munich, Germany; 2 Department of Urology, University Hospital of Munich, Munich, Germany

**Keywords:** Urological manifestations, Solid-state lasers, Characterization and analytical techniques

## Abstract

In order to evaluate the technical adaptability of a type of disposable endoscope compared to reusable flexible endoscopes, *in vitro* and *in vivo* studies were conducted. A disposable digital ureteroscope (“chip on tip”) and two reusable endoscopes were investigated with respect to spatial resolution, geometric distortion in air and water the maximum. Additionally, the clinical performance of the disposable device was tested during clinical procedures (n = 20). The disposable endoscope showed an optical resolution of 6.72 lines/mm at 10 mm distance, similar to the other devices. In comparison, the disposable endoscope showed a barrel-shaped image distortion in air of −24.2%, which is in the middle range, but was best under water (−8.6%). The bendability of 297° (275 µm fiber) and 316° (empty channel, 1.5 F basket) and the maximum irrigation (1 m: 58.1 ml/min, 2 m: 91.9 ml/min) were convincing. Clinically the maneuverability was very good in (13/20), good or satisfactory in (7/20). Visibility was evaluated as very good in (11/20), just in (1/20) either satisfactory or sufficient. The consistency of visibility was not affected in (19/20). In all cases there were no adverse events. The technical examination and clinical application of the disposable endoscope are of equal quality compared to reusable devices. Disposable endoscopes can be an alternative to reusable devices, but economic aspects such as reduction of repair costs, sterilization effort and additional waste must be taken into account.

## Introduction

Since the introduction of a working channel for instruments with a small diameter and fluid-irrigation and the improvement of tip deflection, light transmission and the implementation of digital platforms in endoscopes of the fourth generation, flexible ureteroscopy has changed significantly over the last decades. All these improvements led to an increase of the stone free rates e.g. in patients with stone disease in the upper urinary tract and a decrease e.g. of the operating time, the number of re-intervention and of complication rate^1^. Albeit the significant advancements in the technology of the endoscopic instruments there remain ongoing concerns regarding the durability, longevity, possible contamination, and the cost-effectivity of reusable ureteroscopes.

Ureteroscopic Ho:YAG laser lithotripsy is a preferred method for treatment of ureteral and renal stones^2,3^. Therefore flexible ureteroscopes (FURS) are introduced to the urinary tract and guided to the stone’s site where laser application and hence calculi destruction takes place^4^. Different kinds of flexible multi-use endoscopes are available on the market: conventional FURS, in which the image is delivered via a fibre bundle, FURS with attached camera system for image acquisition, and novel digital FURS with “chip on the tip” technology^5,6^. During the last few years huge improvements proceeded to image quality, durability, irrigation flow and reduction of the shaft diameter^3,4,7^, and thus evolved the ureteroscopic Ho:YAG laser lithotripsy to a safer and highly efficient method for calculi destruction^2,8^. Actually complications like ureteral perforation or stricture formation could be reduced to 5% and 2%, respectively^9^. Depending on the stone’s location an overall stone free rate of 81% to 94% could be achieved via ureteroscopic treatment. A further approach in the development of endoscopic devices is the introduction of disposable, single-use endoscopes^8,10,11^, whereby additionally the calculated total procedure expense of the clinical intervention, e.g. laser lithotripsy, could become more cost effective. The reason is that sterilization or repair procedures are not required for disposable devices, which are one of the main expense factors^12–15^. In the present study a disposable flexible endoscope (PU3022A, Zhuhai PUSEN Medical Technology, Zhuhai, Guangdong China)^16^ was systematically tested with special interest in the image quality in terms of spatial resolution and distortion, as well as the maximal bending capability and the irrigation flow. Data were compared to published results^5,17^ and two reusable flexible endoscopes currently used in the operating room, a video FURS with attached camera system (FLEX X^C^, KARL STORZ SE & Co. KG, Tuttlingen Germany) and a fiberoptic FURS device (FLEX X^2S^, KARL STORZ SE & Co. KG, Tuttlingen Germany). Additionally destruction tests were performed with one single-use device, whereby the application fibre was withdrawn from +10 mm distance in respect to the working channel outlet to (−2 mm) inside the endoscope. In addition to lab investigations the application of the single-use device was tested during clinical interventions.

## Material and Methods

### *In vitro* evaluations

All experiments were performed to test the single-use ureteroscope (PU3022A, 2nd-Generation production year of 2018, Zhuhai PUSEN Medical Technology, Zhuhai, Guangdong China) in terms of: spatial resolution, geometric distortion, water irrigation and bending capability. The data was compared to a reusable fibre optic (FLEX X^2S^, KARL STORZ SE & Co. KG, Tuttlingen Germany) endoscope and a reusable video ureteroscope (FLEX X^C^, KARL STORZ SE & Co. KG, Tuttlingen Germany) performing the same tests.

#### Spatial resolution

The spatial resolution was determined on endoscopic images of a USAF standard (USAF 1951, Thorlabs GmbH, Dachau, Germany) taken at distances of 3, 5, and 10 mm between the distal end of the endoscope and the target. Subsequent the smallest resolvable group and element number of the USAF-standard was identified and thereby the maximal resolvable lines per mm calculated^18,19^. To get information about the reproducibility of single-use ureteroscopes ten different devices were tested plus two reusable endoscopes. Mean values and standard deviations were calculated.

#### Geometric distortion

**Figure 1:**
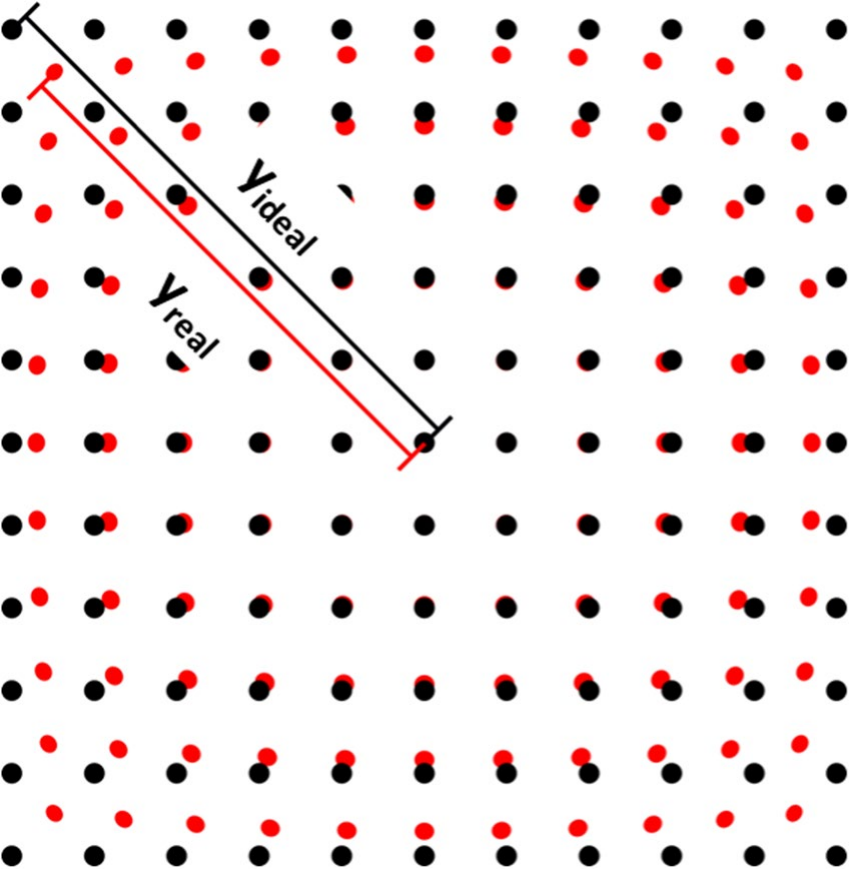
Schematic of barrel shaped distortion of a dot pattern. Black pattern represent the undistorted (ideal) pattern, while the red one stands for the barrel distorted (real) pattern. The diagonal lines y_ideal_ (black) and y_real_ are illustrated for upper left quadrant of the image.

**Figure 2:**
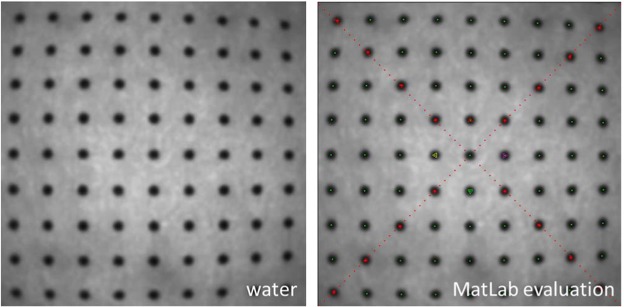
Exemplary images of a dot pattern for distortion measurements in 10 mm distance to the distal end of the single-use endoscope in water and illustration of the software based detection algorithm (green: detected dot centres, red: diagonal dot centres relative to image centre) for subsequent comparison of the paraxial image height (y_ideal_) with the real distorted image height (y_real_).

The geometric distortion was determined by means of images of dot-grids taken in air and under water at distances of 5 mm and 10 mm. This experiment was performed on four single-use devices and both reusable endoscopes. The geometric distortion along the relative image height was calculated by a specifically developed algorithm (Matlab R2014b, The MathWorks Inc., Natick, MA, USA) which implemented the definition of the geometric distortion V, a function of the image height, as $V=\frac{{y}_{real}-{y}_{ideal}}{{y}_{ideal}}$^20^. This formula compares the paraxial image height (y_ideal_) with the real distorted image height (y_real_) for the diagonals (Fig. 1, marked in red in Fig. 2 right) of each image.

The four resulting curves were each fitted by a third order function^21^, reflecting the dependency of the distortion on the field of view respectively the image height and the maximum distortion V_max_ was defined as the arithmetic mean of the calculated distortion at full image height of the diagonals. A negative signed V_max_ corresponds to a barrel shaped distortion, a positive one for pincushion distortion^18,20,22^. In Fig. 2 (left) the image of a dot pattern in 10 mm distance under water is shown, on the right the green marked dots and diagonals (red) due to the detection algorithm are shown.

#### Water irrigation

**Figure 3:**
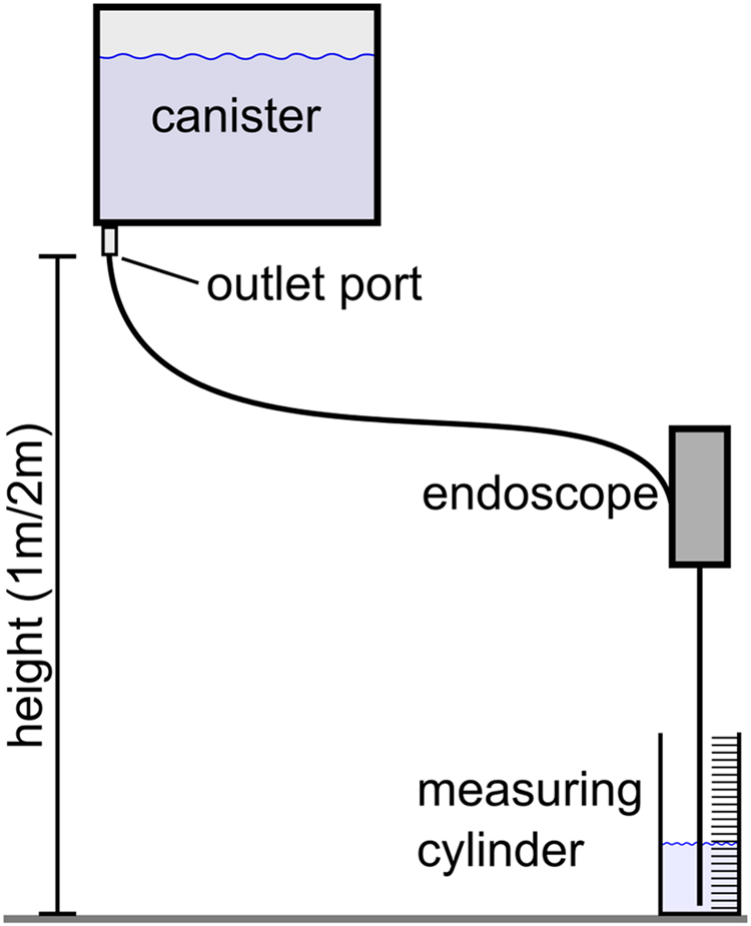
Schematic of the water irrigation measurement set-up.

The experimental set-up to determine the water irrigation is shown in Fig. 3. Mimicking the clinical situation, the outlet-port of a water filled canister was positioned in defined height (h = 1 m and 2 m) related to the outlet-port of the working channel of the endoscope. Three different situations were included in this experiment: empty working channel or filled with either a 275 µm laser fibre (Gentle Flex, Dornier Med Tech Laser, Germany) or a 1.5 F wire basket (NCircle Nitinol basket, Cook, Germany), respectively. The experiment was repeated three times for each combination of height and situation. The water irrigation value was measured in [ml/min]. This experiment was performed on 10 single-use and both reusable instruments, means and standard deviations were calculated.

#### Deflection capability

**Figure 4:**
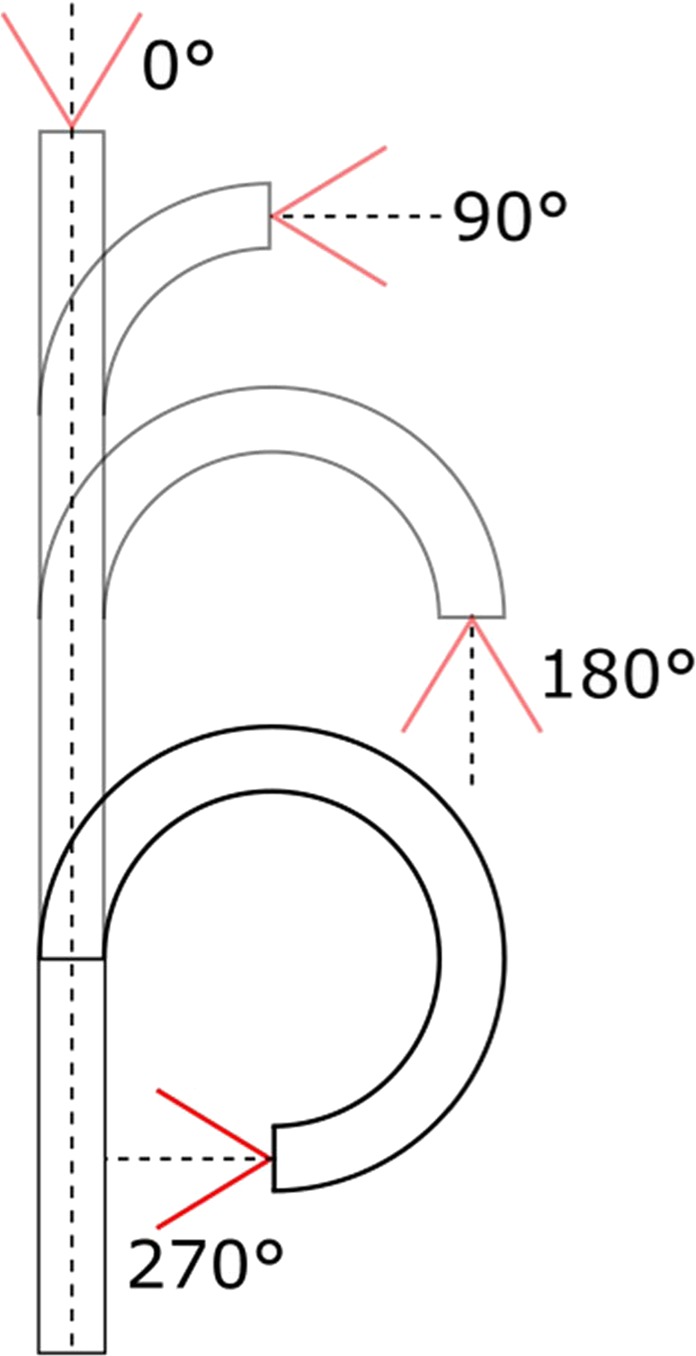
Schematic representation of the bending angle from 0° to 270° by moving the flexible endoscope tip.

Deflection measurements were performed with an empty working channel as well as using the aforementioned 275 µm fiber or 1.5 F wire basket introduced into the working channel. Therefore the distal end of the endoscope was put flat on an even surface, subsequent the outlines of the endoscope tip were traced and the maximum bending angle was measured by means of an angle meter (Fig. 4). This experiment was performed on 10 single-use and both reusable instruments, means and standard deviations were calculated for the single-use device.

#### Destruction test

**Figure 5:**
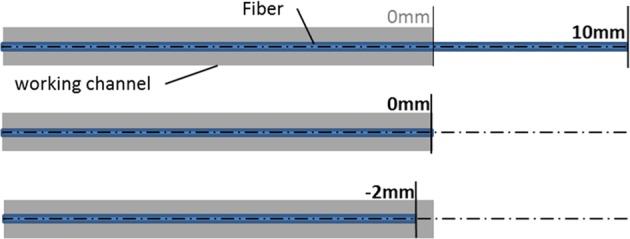
Destruction experiment set-up; the fibre is withdrawn in the working channel in 1 mm steps from 10 mm (fibre tip to distal end of working channel) to −2 mm. Exemplary the fibre positions for 10 mm, 0 mm and −2 mm are shown.

The destruction tests were performed to estimate the damage threshold, hence the robustness, of the camera chip on the tip at the distal end of the single-use endoscope due to laser application near to it. Therefore the fiber was withdrawn in 1 mm steps from 10 mm distance until the fiber tip was even to the working channel outlet (Fig. 5). In every distance the laser (experimental Ho:YAG laser, λ = 2.1 µm) was fired for 10 seconds with 1 J per pulse, 10 Hz repetition rate and 1 ms pulse duration. During and after every laser application the image was observed, whether there were changes in image or illumination quality. Additionally the laser was fired 60 seconds at position 0 mm (fiber tip even to the working channel outlet) and 2 mm inside the channel. These experiments were performed in air and under water (submerged in an aquarium) with one single-use device, no water irrigation was induced to mimic worst case scenario. In the very end destruction tests were performed, whereby the laser was fired with the same setting as mentioned above at position 0 mm for 5 minutes and at position −2 mm until the endoscope tip was destroyed.

#### Statistical evaluation

Statistical evaluation of the data (mean, standard deviation, significance) was performed by using Sigma Plot (V.11.0, Systat Software GmbH, Erkrath, Germany). For multiple group significance calculation the one way ANOVA (Holm-Sidak) method was used.

### Investigation of clinical performance

The clinical applicability of the disposable endoscope was performed as a supplement to the technical investigations. A small group of patients (n = 20) was taken from a large study in order to obtain a reference value for the clinical applicability. These data are only intended to emphasize the subjective assessment of the respective surgeon when using the disposable endoscope and are only to be seen as a first assessment due to the small number of test persons. This assessment makes sense, as the laboratory results are extended by the direct relation to clinical practice work. The results of a large-scale study to evaluate the clinical applicability of the PU3022 will be presented in a follow-up publication.

After approval by the ethical committee of the LMU Munich, Germany, Project number 17–533; WHO-registration: DRKS00017646 [Date of registration: 02/08/2019] (http://www.drks.de/DRKS00017646) and written consent by the patients suffering from stone disease in the upper urinary tract, the single-use ureteroscope PU3022A was tested during 20 interventions. The operations were conducted from two independent operators. Primary endpoint of this feasibility study was to evaluate the manoeuvrability, the visibility, the consistency of the visibility during the whole intervention, the irrigation flow and the endoscopes fatigability. For evaluation a score grading from 1 to 6 (1 = very good, 2 = good, 3 = satisfying, 4 = enough, 5 = deficient and 6 = inadequately) was used, commented by the physician immediately after intervention. Secondary endpoint was the stone free rate, the number of necessary re-intervention, the number of stone recovery and all over operating time.

## Results

### *In vitro* experiments

The basic technical data and the results gained from the measurements with the single–use and the reusable ureteroscopes are listed in Table 1.

**Table 1:** Summary of technical and experimental results (mean + −stddev) of the single use and reusable ureteroscopes.

		PU3022A Single-use	Video FURS Reusable	Fiberoptic FURS Reusable
**Technical data**				
Platform		Digital	Digital	Fiberoptic
Reusable		No	Yes	Yes
Shaft diameter		9.5 F	8.4 F	7.5 F
Working channel		3.6 F	3.6 F	3.6 F
**Optical characteristics**				
**Resolution [lines/mm]**				
10 mm		6.72 ± 0.99	5.66	2.00
5 mm		12.02 ± 0.90	8.98	4.00
3 mm		16.39 ± 0.78	16.00	5.66
**Distortion [%]**				
air	10 mm	−24.3 ± 0.3	−27.6 ± 0.2	−35.7 ± 0.4
	5 mm	−23.2 ± 0.6	−28.4 ± 0.1	−34.5 ± 0.4
water	10 mm	−8.6 ± 0.2	−15.2 ± 0.2	−20.2 ± 0.9
	5 mm	−8.9 ± 0.6	−15.4 ± 0.2	−20.8 ± 0.6
**Deflection [°]**				
empty		316 ± 4	256	248
275 µm fibre		298 ± 10	242	223
wire basket		316 ± 6	245	245
**Flow rate [ml/min]**				
1 m	empty	58.1 ± 2.7	40.5 ± 0.3	36.5 ± 1.1
	275 µm fibre	30.4 ± 1.5	20.3 ± 0.6	19.9 ± 0.5
	wire basket	24.7 ± 1.3	16.5 ± 0.8	14.9 ± 0.9
2 m	empty	91.9 ± 7.4	72.4 ± 1.6	67.9 ± 0.7
	275 µm fibre	50.4 ± 2.4	36.2 ± 0.6	34.9 ± 0.5
	wire basket	41.3 ± 1.5	28.2 ± 0.3	30.5 ± 0.7

The evaluation of the maximum optical resolution at 10 mm distance showed that the image quality of the single-use device (6.72 lines/mm) is comparable to the Video FURS device (5.66 lines/mm) for all distances to the USAF target and, taking the standard deviation into account, also similar in comparison to the literature value^5^ of the single use-LithoVue (7.13 lines/mm). The optical resolution of the fiberoptic FURS exhibited a value of 5.66 lines/mm, which is comparable to the CobraSystem (4.00 lines/mm)^5^. In the resolution measurements, the values for the PU3022 were always significantly different (p < 0.001) to the other devices, the only exception was the comparison with the video FURS at a distance of 3 mm (p = 0.378). The performed experiments for all distances between target and endoscope tip also show that the single-use endoscope in comparison to the video FURS showed similar spatial resolutions while the fiber optic endoscope resulted in reduced spatial resolution.

The image distortion value at a distance of 10 mm in air of −24.3% for the PU3022A was lower than for the other devices (video and fiberoptic FURS) tested in this investigation. In comparison to the devices investigated in^5^ the LithoVue (3.60%) was superior followed by the Flex Xc (22.60%) and the COBRA (16.70%). The distortion measurements in this study showed significantly different values for barrel distortion in both air and water for all devices (p < 0.001). In the study presented here the geometric distortion of the images was evaluated, hence these values are signed (positive: pincushion shape, negative: barrel shape) according to conventions in optical design literature^18,20,22^. In contrast to the values presented by Dale *et al*.^5^, where a positive signed value corresponds to barrel shaped distortion. Additionally geometric distortion values in water were determined, the PU3022A showed distortion values of −8.9% (distance 5 mm) and −8.6% (distance 10 mm), the video FURS (5 mm: −15.4%; 10 mm: −15.4%) and the fiberoptic FURS (5 mm: −20.8%; 10 mm: −20.2%).

The maximum deflection angle of 316° in both directions, achieved for the single-use ureteroscope with an empty working channel was superior to all other measurements and devices. Even if the working channel was filled with the 275 µm fibre and the 1.5 F basket deflection angles of 297° and 316°, respectively, could be reached. The comparison of the single-use instruments in terms of deflection showed 20–40° more bending possibility. In comparison to the reusable instruments, the deflection of the single-use device showed about 55–75° more flexibility depending on the filling of the working channel.

Concerning the irrigation flow with an empty working channel placed at 1 m canister height, the LithoVue^5^, FlexXc^5^ and Fiberoptic FURS and Video FURS revealed relatively similar mean values of 40.3 ml/min, 38.4 ml/min, 36.5, ml/min and 40.5 ml/min respectively, while the CobraSystem^5^ showed the lowest irrigation (28.8 ml/min) and the significant mean value (p < 0.001) was determined for the single-use ureteroscope with 58.1 ml/min. Inserting a fibre reduced the irrigation flow less than the insertion of a wire basket, which was also shown by Dale *et al*.^5^. For 2 m canister height the PU3022A showed superior irrigation, p < 0.001, (empty: 91.9 ml/min, fiber: 50.4 ml/min, basket: 41.3 ml/min) in comparison to the Fiberoptic FURS (empty: 67.9 ml/min, fiber: 34.9 ml/min, basket: 30.5 ml/min) and Video FURS (empty: 72.4 ml/min, fiber: 36.2 ml/min, basket: 28.2 ml/min).

The comparison of single-use and reusable endoscopes in this study showed that the single-use device reached between 45–65% more irrigation capacity at 1 m canister height, and between 30–50% more irrigation capacity at 2 m canister height, in dependency of the filling of the working channel. Thus this investigation exhibited that the single-use PU3022A showed superior irrigation flow values, while the Video FURS and fiberoptic FURS revealed nearly similar flow rates compared to each other.

**Figure 6:**
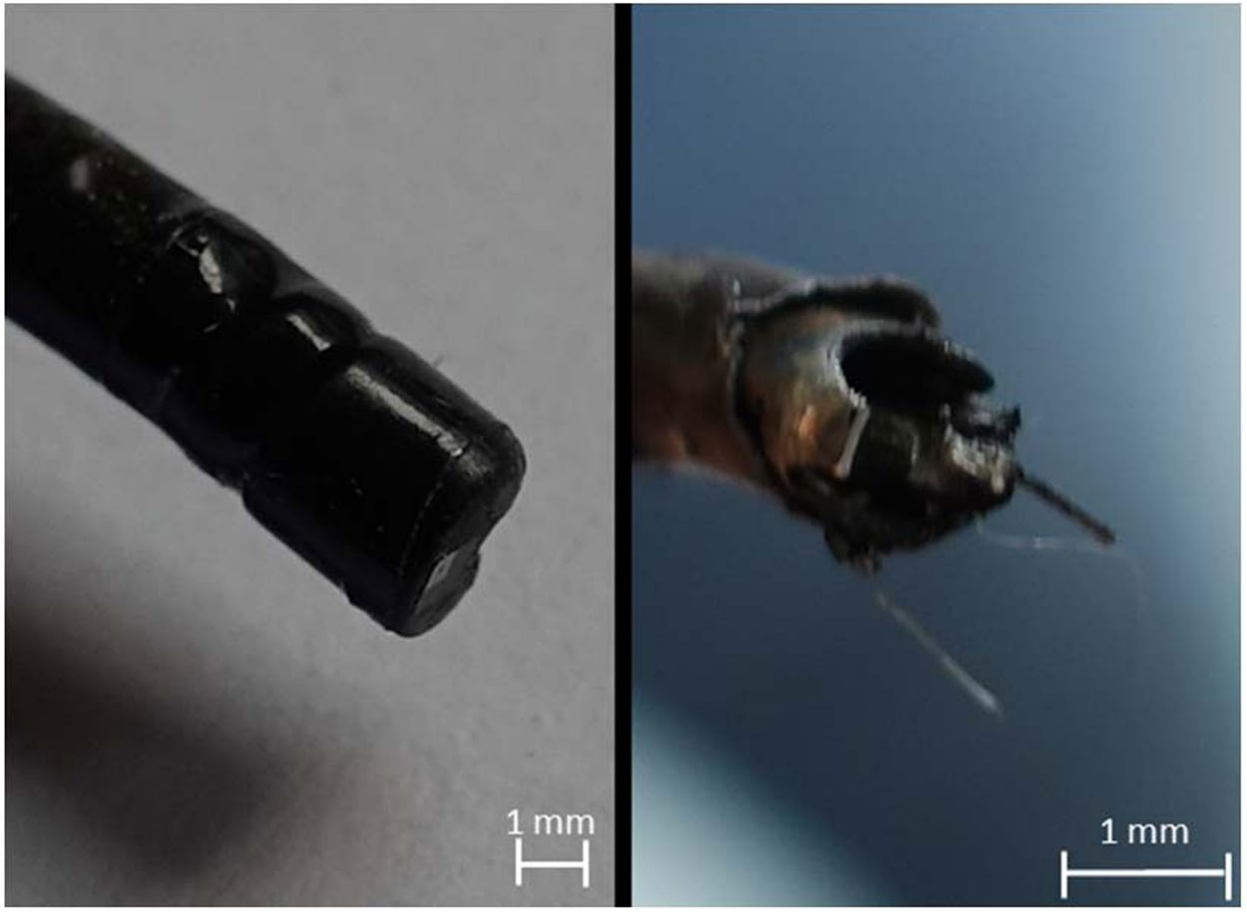
Endoscope tip before laser application at fiber position −2 mm (left) and after firing the laser for 28 seconds (right).

Concerning the robustness test of the camera chip, the PU3022A showed a high damage threshold. By submerging the endoscope in water, without irrigation flow, and firing the laser at every fiber tip to working channel position (10 mm to −2 mm) for 10 seconds, no deterioration in image and illumination quality could be noticed. Even when firing for 5 minutes in distance 0 mm (fiber tip even to working channel outlet) no impact on image and illumination quality was determined. The experiment was also performed in air (without irrigation flow). For distances from 10 mm to 0 mm no effect could be observed, even when firing the laser for 5 min at position 0 mm. Destruction of the endoscope tip, as shown in Fig. 6, was just possible by firing the laser inside the working channel (−2 mm fiber position) for 28 seconds. At around 24 seconds the image became blurry, at 28 seconds the image vanished completely.

### Clinical performance

Patients’ characteristics and the procedures conducted using the single-use ureteroscope are summarized in Table 2. The mean age was 52 y (17–69 y), there were 6 female and 14 male patients (n = 20), and the mean body-mass-index (BMI) was 51 (17–69). The total number of stones found was 23 with a mean stone diameter of 0.9 cm (0.5–1.6 cm). Stones were localised in the lower pole of the kidney (14/23) and 8/23 in the upper/mid pole of the kidney, in the pelvis no stones were present. Three patients suffered from multiple stones, while in one patient no renal or ureteral calculi could be found after inspection.

**Table 2:** Summary of patients demography, stone and intervention data.

Patients demography	
Number of patients	20
single stone	17
multiple stone	3
Age [year] mean (range)	52 (17–69)
Sex (female/male)	6/14
BMI mean (range)	51 (17–69)
Preoperative stent	15/20
**Stone data**	
Total number of stones	23
Mean Stone diameter [cm] mean (range)	0.9 (0.5–1.6)
Stone localisation	
upper-mid pole	8/23
lower pole	14/23
no stone	1/23
**Intervention data**	
Access sheath	14/20
Lithotripsy (Ho:YAG)	16/20
Basket (1.5 F)	18/20
Mean Stone removal	8 (0–30)
Postoperative Stent	14/20

All clinical interventions could be performed without any complication related to the endoscopic device, no additional or exchange endoscopes were necessary during treatment. None of the patients needed active ureteral dilatation to facilitate the procedure. In 16/20 of the interventions a Ho:YAG laser (Medilas H20, Dornier MedTech GmbH, Weßling, Germany) with laser parameter of 1.5 J per pulse and repetition rate of 10 Hz was used for stone fragmentation using a 275 µm application fiber (Gentle Flex, Dornier Med Tech Laser, Germany). Stone removal was performed by means of a 1.5 F zero tip basket (NCircle Nitinol basket, Cook, Germany). The mean number of stone recovery was 8 (0–30). The mean overall operation time was 60 min (34–97 min) and the mean time of single-use endoscope intervention was 38 min (12–76 min), respectively. There were no adverse events in all cases and a stone free rate after intervention in 15/20 patients could be achieved.

In 20 interventions, the maneuverability was in (13/20) very good, in (5/20) good and in (2/20) satisfying. In (11/20) the visibility was marked as very good, in (7/20) as good, in (1/20) as satisfying and in (1/20) as enough. The consistency of the visibility was not affected in (19/20) while in one case a decrease during the operation could be realized. The irrigation flow was constant in all procedures.

Five of the patients were pre-stented and in 14% of the patients a ureter-catheter was inserted for postoperatively care. In (14/20) of the interventions, a 12 F ureteral access sheath (Flexor® 12 F, COOK MEDICAL LLC, Bloomington, IN, USA) was placed to the level of the ureter/ureteropelvic junction. In (6/20) of the cases, the instrument was passed in a monorail fashion over a guidewire.

## Discussion

In a recently published prospective study from Legemate *et al*.^23^, the durability of the latest generation of digital and fiberoptic reusable flexible endoscopes were evaluated. The durability was limited to 27 uses (in summary about 14 working-hours), the damage of the shaft was the most important limitation^23^. These findings are in agreement with the results from other studies published in literature, in some studies the lifetime is even shorter depending on whether it is a fiberoptic or digital instrument^24,25^. These authors recommend not to force the deflection of the instrument during an operation to extend the longevity of an instrument. Nevertheless this conservation due to lower deflection of the device might influence the quality of the operation outcome. With the introduction and usage of single-use instruments, the operator can force the deflection without considering the risk of damage, which may have any effects in future interventions. Carey and colleagues evaluated, that the lifetime of new flexible fiberoptic endoscopes of 40–48 hours of usage, decreased to 11.1 hours after the first repair^1^. In another study from Tosoian *et al*., the average lifetime of a reusable instrument was around 20 interventions, in case of maintenance required this costed in average $605 per use, respectively treatments performed during the lifespan to the next repair cycle^15^. Even if there are recently published studies about the comparison between the costs of reusable instruments and single use instruments, every institution has to calculate if the shift from reusable to single use endoscopes obtains an economic advantage. The data show, taking all factors which influence the costs of reusable endoscopes into account, e.g. costs for labour, consumables and repair, the investment in reusable and single use instruments seems to be comparable^13^.

Another limitation of reusable endoscopes is the risk of contamination. Even if the reprocessing underlies a high quality and standard, it is debatable if the reusable instruments are “100%” clean. Even if there are lacking data about the risk of ureteroscopes and cross infection, there is some evidence that brand new instruments can be contaminated with hemoglobin and high protein levels after initial reprocessing, although no contamination was found before reprocessing. Ofstead and colleagues found, that the risk of contamination was 100% (microbial growth 13%, adenosine triphosphate 44%, hemoglobin 63%, and protein 100%) and those levels exceeded benchmarks for clean gastrointestinal endoscopes^26^. Chang *et al*. reported about the outbreak of ertapenem-resistent Enterobacter cloacae urinary tract infections due to a contaminated ureteroscope^27^. The risk of cross infection can be excluded when using a single use endoscope and could influence the decision whether to prefer using a single-use endoscope.

The present experimental investigation compared the single-use endoscope PU3022A (Zhuhai Pusen Medical Technology Company Limited, China) with reusable digital flexible and reusable fiber optic endoscopes, both from the latest generation.Recently there was an *in vitro* study published from Marchini *et al*.^17^, comparing a single-use ureteroscope (UE3011, Zhuhai PUSEN Medical Technology, Zhuhai, Guangdong China) identified as first generation device with the single use ureteroscope LithoVue (Boston Scientific, Marlborough, Massachusetts, USA) and the Flex-X^2S^ ureteroscope (KARL STORZ SE & Co. KG, Tuttlingen, Germany). The comparison was performed in a similar manner as in this study. In the study^17^ the LithoVue device was superior to the comparison instruments taking the optical resolution, the field of view, the deflection capacity, and the irrigation flow into account.

Concerning the investigated parameters the PU3022A is comparable to other currently used single-use (LithoVue) or reusable FURS (COBRA, Flex-Xc)^5^ and is a promising tool for the surgeon as the preliminary results of the *in vivo* feasibility evaluation (n = 20) showed. Based on published data, one can derive a great technical improvement from 1^st^ generation UE3011 chip-on-the-tip devices to 2^nd^ generation PU3022A regarding image quality and resolution, 2.52 lines/mm versus 6.72 lines/mm, deflection (250° vs. 316°) and irrigation flow rate (52 ml/min vs. 58.1 ml/min)^17^. The increased flow rate of the PU3022 in the laboratory experiments cannot be explained by the larger shaft diameter (9.5 Charr) alone, since the working channel diameter in all three ureteroscopes was 3.6 F. The shaft diameters of FLEX X^C^ and FLEX X^2S^ are smaller with 8.4 F and 7.5 F. It can only be assumed that the working channel material is somewhat more elastic and therefore more space was available overall, even when surgical tools were inserted in the working channel. Reproducible laboratory experiments definitely showed a higher flow rate for the PU3022 in contrast to the other ureteroscopes. In terms of clinical application, the 20 operations performed did not show any adverse effects due to the larger shaft diameter of the PU3022. The 20 surgeries could be performed routinely, and even when using an access sheath, normal OR operation was not affected by the larger shaft diameter. Since the clinical investigations in this study were only intended to provide a first assessment of the feasibility of this endoscope type, the effects or impairments of using this endoscope will only be discussed in detail and in a statistically validated manner in the following publication of the complete study. The geometric distortion of the PU3022A, the video FURS and the fiberoptic FURS was evaluated in air and in water, whereby the underwater experiments may be of greater value, since endoscopes for urolithiasis treatment are usually used in aqueous media. Differences in air to water values can be explained by the different refractive-index matching. The correction of distortion in endoscopic devices^28–31^ and thus image quality improvement may also be a future development based on data sets like the here presented. As can be derived from the obtained results the geometric barrel distortion is smaller under water compared to the air experiment. It can be assumed that the optical system was optimized for underwater operation. All in all the presented distortion values are higher than those of Dale *et al*.^5^. Unfortunately, as the definition and evaluation of the distortion wasn’t transparent, a direct comparison to Dale *et al*.^5^ is not possible. For comparison the definition of distortion is of utmost importance because the TV-distortion describes the bending of a horizontal line close to the edge of the image^18^ while the geometric distortion describes the change of the magnification over the image height^22^ as a function of the image height. In the present study the geometric distortion definition and evaluation was used. From the published images in^5^ the herein used evaluation procedure cannot be discovered taking additional optical system design literature into account^18,20,22^.

Compared to the results in the work of Dale *et al*.^5^, the PU3022A shows the third highest spatial resolution of 6.73 lines/mm at a distance of 10 mm after the Flex-Xc (8 lines/mm) and the disposable LithoVue (7.13 lines/mm) and thus surpasses the FURS from this work as well as from Dale *et al*. In terms of image distortion among the compared PU3022A, video FURS and fiber optic FURS in this study, the PU3022A shows the lowest value of −24.3% compared to −27.6% and −35.7%, respectively. Nevertheless, as explained above, the distortion value measured in water is more important because it reflects the environment in which an endoscope intended for urological procedures is used. A direct comparison with the values from Dale *et al*. is not possible for the reasons mentioned above. The PU3022A shows the highest flow rate of all compared endoscopes in this study at 58.1 ml/min (empty working channel, 1 m height) and Dale *et al*., despite the same working channel diameter of 3.6 F as all other compared endoscopes, except Cobra with Dual 3.3 F, which shows the lowest flow rate (28.8 ml/min). In deflection, the PU3022A with 316 ± 4° exceeds the disposable LithoVue with 276°^5^, even with a 275 µm fiber inserted into the working channel, 298 ± 10° can be achieved, compared to 274° of a LithoVue with a thinner 200 µm fiber inserted. In general, the PU3022A appears to outperform in most of the parameters compared, l*et al*one in the spatial resolution, which at 6.72 lines/mm is still in the upper range (between 2 and 8 lines/mm) of the endoscopes compared in this study and Dale *et al*.

The present study showed also some mechanical advantages of the single-use instrument in comparison to the reusable endoscopes. The single-use PU3022A showed more bending flexibility and improved deflection of up to 75° at the distal end and additional an increased irrigation flow rate by 30–70%, both depending on the device (fiber, basket) in the working channel. Although the single-use device is made as a “low-cost” version, the production reproducibility is overall good as can be derived from small standard deviations in the inter-sample comparison. This is the base of one further potential advantage of single-use FURS, it is every time a “new and sterile” product having highly reproducible and guaranteed quality in terms of image spatial resolution and distortion, deflection capability and irrigation flow, etc. Also in terms of robustness of the camera chip the PU3022A showed convincing results, however more experiments have to be performed to gain reliable information. The comparison devices in this study, the video FURS and fiberoptic FURS, both were clinical used for several times before undergoing this investigation and thus these were not new devices. This “reusable status” may influence the mechanics and optics, hence the outcome in the experiments performed, whereas the PU3022A endoscopes were always brand–new. Nevertheless these devices are still used in the operation room and thus exactly reflects the daily situation in clinical practice, using a reusable instead of brand-new instrument to clarify the upper urinary tract, to get histology, to treat tumors and strictures and to treat patients with stone diseases. Even if the reusable instruments underlie a strict control after the reprocessing procedure including e.g. the sterilization, the high quality of reusable instruments cannot be guaranteed over their whole lifetime. With the availability of single-use ureteroscopes on the market, a new era in the field of endourology has begun. Whether it is an advantage or not, has to be evaluated in further studies. First step is to compare the new instruments and the instruments used in daily clinical practice in standardized experiments as performed in this study. The second important step is to evaluate the instruments in prospective randomized clinical studies. The presented clinical feasibility testing of the single-use instruments shows that all clinical endourological procedures could be performed without any complications. Since this is a first feasibility study on a small group (n = 20), the point system presented for evaluating the subjective assessment of the surgeon cannot be compared with statistically proven assessments from large cohorts. This was not the intention at all, but the clinical data should confirm laboratory tests and thus underline the scientific knowledge gained. This is particularly important, as laboratory tests often have little or no relation to the later application. A clinical assessment by experienced physicians, even on a small cohort of 20 probands, is therefore valuable to substantiate the *in vitro* findings. Nevertheless, the medical and clinical applicability will be examined in greater detail in a subsequent publication, which will then contain the total cohort (n = 120). Anyhow, the presented data show that the PUSEN 3022A is a promising tool for endoscopic interventions in the upper urinary tract. Whether it is comparable with the instruments of the newest generation (digital and fiberoptic instruments) has to be evaluated. As the discussion about the advantages of single-use or reusable ureteroscopes is on debate some advantages and disadvantages of single use instruments and reusable instruments are summarized in Table 3.

**Table 3:** Summary single-use FURS vs. re-usable FURS.

Single use FURS		reusable FURS	
+	−	+	−
single use no sterilization process existing infrastructure* (Distribution, no repair facilities)	environmental impact: waste disposal^32–34^	sustainable multi use existing infrastructure* (distribution, sterilization, maintenance)	environmental impact: toxic detergents for sterilization^33–35^
always new and sterile “brand-new” optics/mechanics	“New technology” “digital optics ureteroscopes”, shaft diameter 9.5 F data acquisition	“established technology” optimized optical systems shaft diameter 8.5 F/8.0 F data acquisition	“used status” influences optics/mechanics cross contamination between patients possible^26,27,35^
low acquisition costs and no current expense**			high acquisition costs plus current expense** (maintenance, sterilization)

*The use of both devices depends on the experience of the surgeon and the whole operation team as well as on the infrastructure (sterilization institutions/repair facilities) in the respective country or rather clinics^35^.

**Cost analysis show different outcomes concerning the profitability of a reusable or single use endoscope program, depending on institute size, case numbers and infrastructure^1,13,15^.

## Conclusion

Both the clinical and laboratory evaluation showed markedly high performance for the single-use endoscope, which is comparable to the one of multi-used instruments. Disposable endoscopes may be a reliable and cost effective, but not necessary ecologically friendly^32,33^ approach towards reusable ones^8^.

### Research involving human participants

After approval by the ethical committee of the LMU Munich, Germany, (Project number 17–533) all methods were performed in accordance with the relevant guidelines and the ethical standards laid down in the 1964 Declaration of Helsinki and its later amendments. Informed consent was obtained of all patients prior to inclusion to the study. The single-use ureteroscope PU3022A was tested during 20 interventions on Patients suffering from stone disease in the upper urinary tract. This study is also officially registered with the DRKS (Deutsches Register Klinischer Studien/German register for clinical studies) and can be found in the WHO Meta-registry [registration number: DRKS00017646] (http://apps.who.int/trialsearch/).
